# Therapy Response and Survival among Patients with Gynecologic Tumors Treated with Transarterial Chemoperfusion and Transarterial Chemoembolization

**DOI:** 10.3390/medicina60101585

**Published:** 2024-09-27

**Authors:** Thomas J. Vogl, Andreea I. Nica, Christian Booz, Leona S. Alizadeh, Sven Becker, Ibrahim Yel, Teodora Biciusca, Aynur Gökduman, Mirela Dimitrova, Christian Wolfram, Tatjana Gruber-Rouh, John Bielfeldt, Hamzah Adwan

**Affiliations:** 1 Clinic for Radiology and Nuclear Medicine, University Hospital Frankfurt, Goethe University, 60590 Frankfurt am Main, Germany; 2 Department of Gynecology and Obstetrics, University Hospital Frankfurt, Goethe University, 60590 Frankfurt am Main, Germany

**Keywords:** transarterial chemoperfusion, transarterial chemoembolization, cervical carcinoma, ovarian carcinoma, uterine carcinoma

## Abstract

*Background and Objectives*: This study aimed to evaluate the tumor response relating to and survival benefit of transarterial chemoperfusion (TACP) and transarterial chemoembolization (TACE) in the treatment of patients with unresectable gynecologic tumors who are intolerant of or have a suboptimal response to chemotherapy and radiotherapy. *Materials and Methods*: Between January 2000 and October 2023, 75 patients diagnosed with gynecologic tumors underwent 213 TACP and 154 TACE procedures. Of these, 33 patients were treated with TACP, 20 were treated with TACE, and 22 received a combination of both therapies. A retrospective evaluation of local tumor response according to Response Evaluation Criteria in Solid Tumors (RECIST) was conducted, and survival rates were determined using the Kaplan–Meier estimator. *Results*: Of the total 75 patients, 50 (67%) maintained a stable course of disease until the completion of therapy, 10 (13%) had a partial response, 2 (3%) had a complete response following thermal ablation, and 13 (17%) experienced progression. Furthermore, a 6% reduction in the sum of the longest diameters and an 8% reduction in tumor volume were observed. The median overall survival was 16.15 months, while the median progression-free survival was 13.19 months. *Conclusions*: TACP and TACE are potential treatment options for local tumor control in patients with unresectable gynecologic tumors who are intolerant of or show a poor response to chemotherapy and radiotherapy. However, further investigation and adjustment of treatment protocols are required to improve therapy response and survival outcomes.

## 1. Introduction

Gynecologic malignancies are the leading cause of mortality among women worldwide, with cervical, uterine, and ovarian carcinoma representing the most prevalent forms. Other, less common tumors include vulvar and vaginal carcinoma, as well as uterine sarcoma [1,2,3].

Cervical cancer is the most frequent gynecologic malignant tumor [3], ranking as the fourth most frequently diagnosed cancer worldwide and the fourth leading cause of cancer-related mortality among women [4]. GLOBOCAN estimates that 604.000 new cases and 342.000 deaths were caused by cervical carcinoma in 2020 [4]. Risk factors for developing cervical cancer include infection with human papillomavirus (HPV), human immunodeficiency virus (HIV), smoking, and hormonal contraception [5]. Despiteb being considered highly preventable through screening and vaccination programs, cervical cancer remains the leading cause of cancer-related mortality in 36 countries [4]. Notably, it affects many young women, with nearly 50% of patients diagnosed with cervical cancer being under the age of 50 [6]. The primary treatment for early-stage cervical cancer is surgical resection, typically involving radical colpohysterectomy with surgical lymph node staging [7]. However, cervical carcinoma is frequently diagnosed at advanced stages, where the first-line treatment is concurrent chemoradiation (for IB3- to IVA-stage disease), mostly involving platinum-based chemotherapy protocols [6,8].

Endometrial carcinoma is the sixth most commonly diagnosed cancer in women, with 417.000 new cases and 97.000 deaths reported in 2020 [4]. Significant risk factors for developing endometrial cancer include increasing age, obesity, physical inactivity, exposure to exogenous estrogen, insulin resistance, and the use of tamoxifen after breast cancer treatment [9]. Curative surgical treatment is primarily indicated for women with stage FIGO I or IIa disease [10]. This comprises a total abdominal or laparoscopic hysterectomy and a bilateral salpingo-oophorectomy. Furthermore, radiotherapy can be employed as an adjuvant treatment following surgical intervention to prevent loco-regional recurrence [11].

Ovarian carcinoma is the fifth most common cause of cancer-related mortality in women and has the highest mortality rate among all gynecologic malignancies [12,13,14]. Risk factors for developing ovarian cancer are advanced age, genetics, a family history of the disease, hormone replacement therapy, nulliparity, and a high-fat diet [13]. The standard treatment for advanced-stage ovarian cancer involves maximal debulking surgery combined with chemotherapy with a platinum compound (Cisplatin, Oxaliplatin, or Carboplatin) and a taxane (Paclitaxel) [12,14]. However, even after optimal debulking, 60–70% of patients experience recurrence, and long-term survival rates remain low [14,15].

While conventional treatments, such as chemotherapy and radiotherapy, are crucial in managing advanced-stage gynecologic malignancies, they are associated with a range of adverse effects [16,17,18]. For instance, radiotherapy may induce acute radiation toxicity (ART), which typically presents as gastrointestinal, hematologic, and genitourinary toxicity [16]. It has been estimated that approximately 84% of patients diagnosed with cervical cancer who undergo radiotherapy experience ART [16]. In addition, platinum-based chemotherapy regimens have a high rate of inducing dose-limiting toxicity [17]. Of these toxicities, neurotoxicity is the most significant systemic side effect of oxaliplatin, while myelosuppression and nephrotoxicity are the most significant side effects of carboplatin and cisplatin, respectively [18]. Recent advances in interventional radiology have led to the development of minimally invasive procedures designed to replace conventional treatments for tumor growth control while minimizing the systemic adverse effects associated with chemotherapeutic agents [10,19]. Initially employed in the treatment of hepatic tumors, transarterial chemoperfusion (TACP) and transarterial chemoembolization (TACE) are designed to administer chemotherapeutic agents directly into the arteries supplying a tumor, with additional embolization and subsequent tumor necrosis in the case of TACE [10,19,20]. Over time, their application has been extended to gynecologic malignancies, as several studies have demonstrated the efficacy of TACE and TACP in the management of cervical cancer [10,21,22]. Song et al. observed that patients with cervical carcinoma who underwent TACE experienced fewer adverse events, such as thrombocytopenia, myelosuppression, and hepatic or renal dysfunction, and had a higher resection rate than those treated with chemoradiotherapy (CRT) [10].

The aim of this study was to assess TACE and TACP as potential alternative treatment options for patients with gynecologic tumors who are intolerant of, exhibit a poor response to, or refuse chemotherapy and radiotherapy and are not suitable candidates for surgery. TACP and TACE were conducted with a palliative intent. Tumor response was evaluated according to the RECIST criteria 1.0 (Response Evaluation Criteria in Solid Tumors, 2000) [23], and overall survival (OS) and progression-free survival (PFS) were determined.

## 2. Materials and Methods

### 2.1. Study Design and Patients

This study was approved by the ethics committee of our university hospital. This retrospective, single-center study included patients diagnosed with unresectable gynecologic tumors who were unable to tolerate, refused, or had a poor response to chemotherapy and radiotherapy. Inclusion criteria for the study were as follows: (1) diagnosis of an unresectable gynecologic tumor; (2) adequate coagulation parameters, as well as renal, hepatic, and thyroid function; (3) undergoing a minimum of two procedures of either TACP, TACE, or both; and (4) over 18 years of age. Exclusion criteria comprised the following: (1) having insufficient follow-up data, (2) having undergone fewer than two procedures, and (3) being under 18 years of age.

A total of 100 female patients treated with TACE and TACP between January 2000 and October 2023 were evaluated. Twenty-five patients were excluded from this study due to a lack of follow-up data or having undergone fewer than two procedures. Consequently, 75 patients (median age, 50 years; range, 24–86 years) were included in the final study group. Of these, 36 patients were diagnosed with cervical carcinoma, 14 were diagnosed with ovarian carcinoma, 11 were diagnosed with endometrial carcinoma, 6 were diagnosed with vaginal carcinoma, 4 were diagnosed with vulvar carcinoma, 2 were diagnosed with uterine leiomyosarcoma, one was diagnosed with uterine carcinosarcoma, and one was diagnosed with stromal sarcoma of the ovary (Table 1). A total of 213 TACP and 154 TACE procedures were collectively performed on these patients. Among the patients, 33 underwent TACP, 20 were treated with TACE, and 22 received a combination of both therapies, with an average of 4 procedures per patient (range 2–16). The majority of patients enrolled in this study had previously undergone chemotherapy and radiotherapy prior to enrollment in the trial. Most patients either discontinued treatment due to intolerable side effects or had a suboptimal response, resulting in disease progression. Some patients opted for transarterial treatment after refusing conventional therapies. Additionally, a subset of patients had previously undergone thermal ablation or surgical resection.

### 2.2. Imaging Modalities

Magnetic resonance imaging (MRI) and computed tomography (CT) scans were conducted at baseline to identify tumor lesions, as well as between therapy sessions to assess tumor response and after the completion of the treatment cycle. Furthermore, a CT scan was performed shortly after each treatment to detect any complications, such as bleeding, as well as to confirm the retention of Lipiodol after performing TACE. The devices used in our department during the study period included the Magnetom Aera, Espree, Prisma fit, and Avanto fit (Siemens, Erlangen, Germany) for MRI and the Somatom Definition AS+ and Somatom Force (Siemens, Erlangen, Germany) for CT. MRI scans comprised native T1- and T2-weighted sequences, along with contrast-enhanced T1 sequences (with a slice thickness ranging from 3 mm to 8 mm).

### 2.3. Treatment Protocol

**Figure 1:**
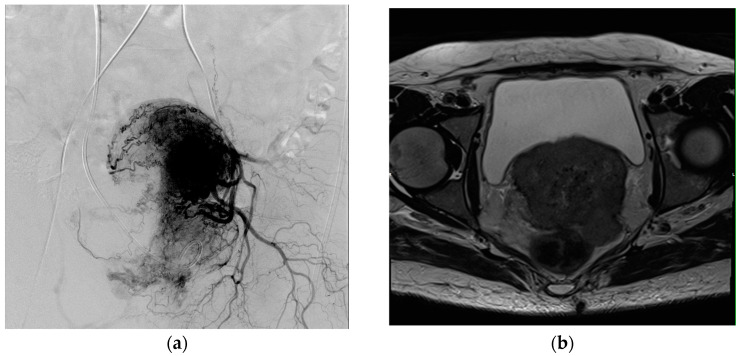
(**a**) Angiographic image of a transarterial chemoembolization (TACE) procedure in a patient with cervical carcinoma T4N2M1. The catheter was placed in the left internal iliac artery. (**b**) Transverse T2-weighted MRI image of the pelvis, in which the treated tumor is detectable.

In preparation for the procedure, the patients’ latest blood results were evaluated, and the patients were informed of the potential adverse effects and risks associated with the therapy. Informed consent was obtained from each patient. The patients were required to fast for six hours prior to the intervention but were permitted to drink fluids and take medication up to two hours beforehand. To prevent adverse effects, such as pain, nausea, and contrast-related allergies, a combination of analgesic (Pethidine, Panpharma, Trittau, Germany) and antiemetic medication (Granisetron, Fresenius Kabi, Bad Homburg, Germany) as well as corticosteroids (Dexacortin, Jenapharm, Jena, Germany) was administered. TACP and TACE were conducted by an experienced interventional radiologist using the Axiom Artis angiography system (Siemens, Erlangen, Germany). For both procedures, we employed the same vascular approach. The procedure was commenced by administering a local anesthetic, mepivacain (Scandicain, AstraZeneca, Wedel, Germany), followed by the puncturing of the femoral artery and the introduction of a sheath and a catheter via the Seldinger technique. Our department utilizes 4 F femoral sheaths, along with a range of catheters, including Pigtail, Renegade (Boston Scientific, Munich, Germany), Sidewinder, Headhunter, and Cobra catheters (Terumo, Tokyo, Japan). Subsequently, an angiography of the abdomen and pelvis was conducted to assess the vascular anatomy. Subsequently, a catheter was selectively positioned in either the inferior mesenteric arteries or branches of the internal iliac artery, employing either an ipsilateral or crossover technique. The subsequent steps to be performed were determined by the type of intervention. If TACP was performed, chemotherapeutic agents were administered for approximately 20 min. In contrast, TACE, which was primarily employed for patients with hypervascularized tumors, commenced with the injection of a mixture of chemotherapeutic and embolizing substances until a stasis of blood flow in the arteries supplying the tumor was noted (Figure 1). Upon the completion of drug administration, the procedure was concluded by the removal of the catheter and the femoral sheath and the closing of the puncture site using the Angio-Seal system (Angio-Seal™, St. Jude Medical, Saint Paul, MN, USA). A pressure bandage was applied to the puncture site, and the patients were observed for a further six hours.

Therapy was performed in four-to-six-week intervals, predominantly in a palliative setting. In a few cases, the treatment was employed in a neoadjuvant setting to reduce tumor size (sum of the longest diameters—SLD) before tumor ablation or surgical resection. Many patients were subjected to systemic chemotherapy in addition to transarterial treatment. The duration of treatment was adjusted according to the therapy response. Treatment was continued until further tumor growth was prevented or a reduction in SLD was achieved, thereby enabling the patients to become eligible for surgery or ablation. Treatment was discontinued either at the patient’s request or as a result of radiologic and clinical progression. Further therapy options were established in an interdisciplinary meeting.

### 2.4. Medication

The primary treatment protocol for TACP procedures included Mitomycin C (Medac, Hamburg, Germany), Gemcitabine (Gemzar, Lilly, Bad Homburg, Germany), and Cisplatin (Cisplatin Teva, Radebeul, Germany). In some patients, varying combinations of Irinotecan (Campto, Pfizer Pharma, Karlsruhe, Germany) and Bevacizumab (Avastin, Roche, Basel, Switzerland) were administered, depending on their previous chemotherapy regimen. For TACE, the main agents employed were mitomycin C, irinotecan, and cisplatin. The doses of chemotherapeutic agents were adjusted according to each patient’s weight. For embolization, Lipiodol and EmboCept were applied. When using EmboCept, a specialized form of TACE was employed, referred to as DSM-TACE (TACE with degradable starch microspheres).

### 2.5. Tumor Volumetry and Diameter Measurements

**Figure 2:**
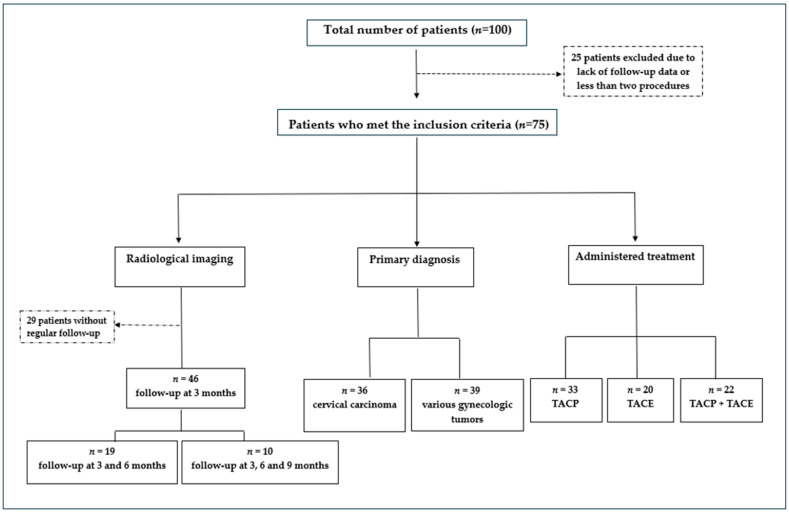
Patients were divided according to three criteria: (1) availability of radiological imaging, (2) primary diagnosis, and (3) administered treatment (TACP = transarterial chemoperfusion). Based on this division, we obtained different patient groups for which we conducted measurements and assessed tumor response and survival data.

Measurements of the tumors were conducted via MRI and CT imaging of the pelvis. Measurements were performed for each patient at baseline and at the last available follow-up CT/MRI scan to provide an overview of the development of SLD in the whole cohort. Additional measurements were taken to compare different groups based on the following criteria: first, the availability of radiological imaging; second, the primary diagnosis; and third, the administered treatment (Figure 2).

Twenty-nine patients did not participate in regular follow-ups and were only evaluated at baseline and the last available follow-up. Consequently, according to the first division criterion, measurements could be performed at regular intervals in only 46 cases. Thus, we obtained three groups: Group 1, the main group, included all 46 patients, for whom measurements were taken at 3 months from the first procedure. Group 2 emerged from Group 1 and included 19 patients, who also underwent additional measurements at 6 months. Group 3 emerged from Group 2 and included 10 patients, who underwent supplementary measurements at 9 months. These measurements were performed at regular intervals to ensure consistency in reporting tumor response and tracking tumor size development over time.

Subsequently, the second division criteria identified two groups: The first group consisted solely of patients diagnosed with cervical carcinoma, with a total of 36 participants, while the other group comprised patients with various gynecologic tumors, with a total of 39 participants. The second group included 14 patients with ovarian carcinoma, 11 cases of endometrial carcinoma, 6 patients with vaginal carcinoma, 4 patients with vulvar carcinoma, 2 cases of uterine leiomyosarcoma, 1 case of uterine carcinosarcoma, 1 one case of stromal sarcoma of the ovary.

Upon categorizing the patients based on the administered treatment, three groups were obtained: The first group included patients who received TACP exclusively (*n* = 33), the second comprised patients who only received TACE (*n* = 20), and the third included patients who received both therapies (*n* = 22).

The measured parameters included the SLDs of all tumor lesions, which were utilized in the assessment of tumor response. Tumor volume (TV) was evaluated through manual slice segmentation. In accordance with the RECIST criteria, a maximum of five lesions per organ and ten overall were evaluated as target lesions, selecting the lesions with the longest diameter and those that allowed for repeated measurements. Lesions with a diameter of ≥20 mm for conventional CT and MRI, and ≥10 mm for spiral CT, were selected and identified as either tumor formations localized in one or multiple pelvic organs, infiltrations of the pelvic bones, or lymph node metastases.

### 2.6. Tumor Response Assessment

**Figure 3:**
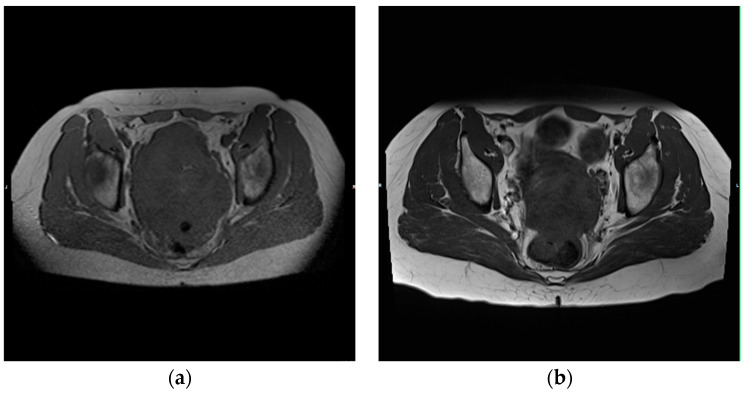
A 47-year-old patient diagnosed with ovarian carcinoma underwent eight TACP procedures. Following the completion of the treatment cycle, the measurements demonstrated a reduction of 34% in the longest diameter. Consequently, the patient exhibited a partial response. (**a**) Transverse T1-weighted image of the pelvis that depicts the tumor prior to the initiation of therapy; (**b**) Transverse T1-weighted image of the pelvis that shows the tumor following the completion of eight TACP procedures, which resulted in a significant reduction in the longest diameter.

The tumor response for each of the previously described patient groups was evaluated. In accordance with the RECIST criteria 1.0 (2000), four distinct types of tumor response can be identified: “partial response” (PR), indicating a reduction of ≥30% in the SLD of the target lesions (Figure 3), using the baseline SLD as reference; “complete response” (CR), meaning the disappearance of all tumor lesions; “stable disease” (SD), defined as a reduction in the SLD of <30% or a growth of <20%; and “progressive disease” (PD), marked by an increase of ≥20% in the SLD from the smallest SLD recorded since the beginning of the study or the occurrence of new tumor lesions.

### 2.7. Statistical Analysis

Statistical analysis was conducted by employing BiAS 11.12 software (Dr. rer. med. Hanns Ackermann, epsilon-Verlag GbR, Frankfurt am Main, Germany), employing the Kaplan–Meier estimator to calculate OS and PFS. The OS was defined as the period between the initiation of the first therapy session and death or last documented contact. The PFS was defined as the time between the initial procedure and disease progression, death, or last contact. The log-rank test was employed to assess for significant variations in survival between patients diagnosed with cervical carcinoma and those with other gynecologic malignancies. In this study, a *p*-value of <0.05 was regarded as statistically significant.

## 3. Results

### 3.1. Overall Tumor Response

After evaluating the tumor responses of all 75 patients included in this study, it was found that 50 (67%) patients maintained SD, 2 patients (3%) achieved CR (after thermal ablation), 10 (13%) had a PR, and 13 patients (17%) experienced disease progression. Consequently, 62 patients (83%) exhibited a response to therapy (SD + PR + CR). The median follow-up time was 7.4 months (range 1–83 months). After performing measurements at baseline and the last available follow-up for all patients, a median SLD of 7.41 cm (range 1.2–20 cm) and a median TV of 129.73 cm^3^ (range 4–620 cm^3^) were observed at baseline. At the final follow-up measurement, the median SLD was 7 cm, and the median TV was 119.67 cm^3^. Overall, a 6% decrease in the median SLD and an 8% decrease in the median TV were observed. Additionally, five patients were deemed eligible for thermal ablation after completing therapy, and one patient was deemed eligible for surgical resection.

### 3.2. Tumor Response Evaluation at 3-Month Intervals

**Figure 4:**
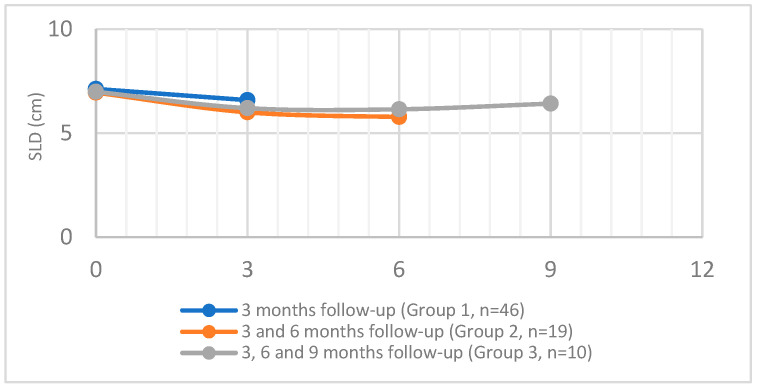
Evaluation of SLD at 3-month intervals for each patient group. Group 1 consisted of 46 patients, for whom measurements were taken at 3 months after the initial procedure. Of the 46 patients, 19 patients (Group 2) underwent measurements at 3 and again at 6 months, and 10 patients (Group 3) underwent measurements at 3 months and again at 6 and 9 months. The *x*-axis represents the 3-month intervals of the follow-up measurements. The *y*-axis represents the SLD in cm.

After dividing the patients according to the availability of follow-up imaging, three groups were formed: The main group (Group 1) included all 46 patients for whom measurements were taken at three months after the initial treatment. Group 2 included 19 patients, who also underwent measurements at 6 months. Group 3 included 10 patients, who underwent additional measurements at 9 months. Accordingly, the following results were obtained (Table 2 and Table 3, Figure 4): In Group 1, the median SLD was 7.12 cm at baseline and 6.59 cm at three months (a 7.44% reduction). Additionally, the median TV was 117 cm^3^ at baseline and 98.48 cm^3^ at the three-month follow-up (a 15.83% decrease).

In Group 2, the median SLD at baseline was 6.95 cm, and the median TV was 117.27 cm^3^. At three months, the median SLD was 6 cm (a 13.67% reduction), and the median TV was 77.99 cm^3^ (a 33.5% reduction). At the six-month follow-up, the median SLD was 5.78 cm (a 3.67% reduction), and the median TV was 68.78 cm^3^ (an 11.81% reduction).

In Group 3, which consisted of 10 patients, the median SLD of the target lesions at baseline was 6.99 cm, and the median TV was 103.4 cm^3^. At three months, the median SLD was 6.2 cm (an 11% decrease), and the median TV was 86 cm^3^ (a 16.8% decrease). At the six-month follow-up, the median SLD was 6.15 cm (a minimal decrease of 0.81%), and the median TV was 80.87 cm^3^ (a 5.97% decrease). At nine months, the median SLD was 6.42 cm (a 4.39% increase), and the median TV of the target lesions was 119.71 cm^3^ (a 48% increase).

**Figure 5:**
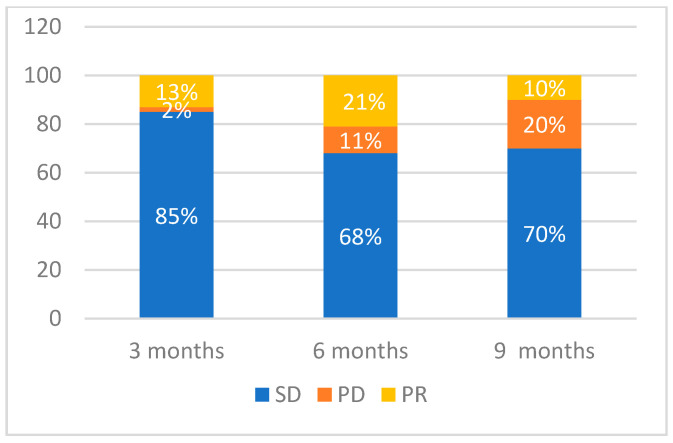
Graphical representation of the tumor response evaluation based on the Response Evaluation Criteria in Solid Tumors (RECIST) at 3-month intervals (SD = stable disease; PD = progressive disease; PR = partial response). A total of 46 patients were evaluated at 3 months. Of these, 39 patients (85%) maintained SD, 6 (13%) displayed a PR, and 1 (2%) experienced PD. At 6 months, 19 patients were evaluated. Of these, 13 (68%) had SD, 4 (21%) had a PR, and 2 (11%) had a PD. At 9 months, we evaluated 10 patients, of which 7 (70%) had SD, 1 (10%) displayed PR, and 2 (20%) experienced progression.

**Figure 6:**
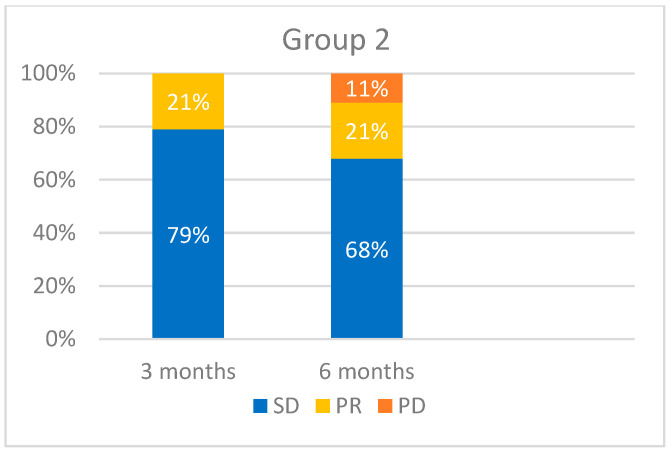
Tumor response evaluation based on RECIST for Group 2 (*n* = 19).

**Figure 7:**
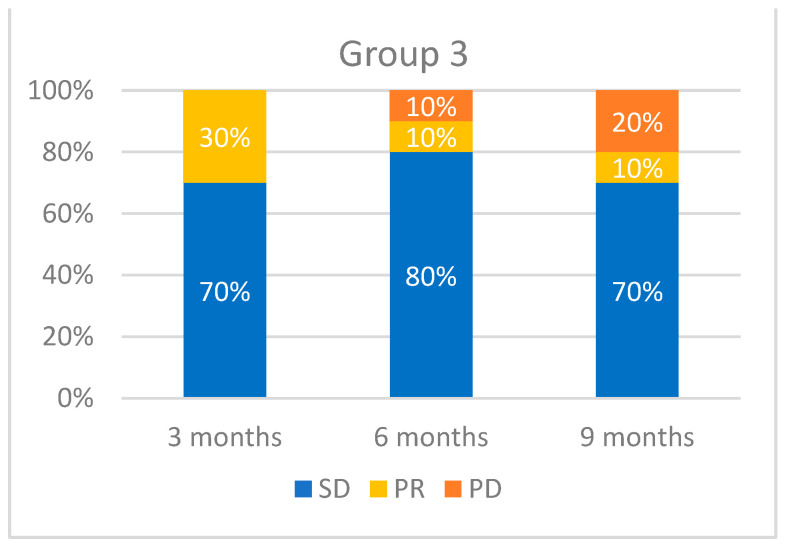
Tumor response evaluation based on RECIST for Group 3 (*n* = 10).

When evaluating tumor response at 3-month intervals, we obtained the following results (Figure 5, Figure 6 and Figure 7): 46 patients were evaluated after 3 months. Of these, 39 patients (85%) maintained SD, 6 (13%) displayed a PR, and 1 patient (2%) experienced PD. At 6 months, 19 patients were evaluated. Of these, 13 (68%) had SD, 4 (21%) had a PR, and 2 (11%) had a PD. At 9 months, we evaluated 10 patients, of which 7 (70%) had SD, 1 (10%) displayed a PR, and 2 (20%) experienced progression. Figure 6 and Figure 7 provide a detailed evaluation of tumor response in Groups 2 and 3.

### 3.3. Tumor Response Evaluation Based on Primary Diagnosis

Upon dividing the patients into two groups based on their primary diagnoses, two groups were identified: the first group consisted of patients diagnosed with cervical carcinoma, totaling 36 patients, while the second group comprised patients with other gynecologic tumors (Table 1), a total of 39 participants.

When assessing tumor response in the first group, we obtained the following results (Table 4): Of the 36 patients, 26 (72%) had SD, 2 exhibited a PR (6%), 1 had a CR (3%), and 7 (19%) displayed PD. Consequently, 29 patients (81%) were therapy responders (SD + PR + CR). In this group, measurements could be performed for 32 of the 36 patients. The median SLD was 7.58 cm at baseline and 7.6 cm at the last follow-up, indicating a minimal increase of 0.26% in the median SLD. The median TV at baseline was 135.2 cm^3^ and 155.7 cm^3^ at the last follow-up, amounting to an increase of 15.16% in the median TV.

The second group yielded the following results (Table 4): Out of 39 patients, 25 (64%) had a SD, 6 (15%) displayed PD, 7 (18%) had a PR, and 1 (3%) achieved a CR. Consequently, 33 (85%) patients in this group were therapy responders. Measurements could be performed for 34 of the 39 patients. The median SLD at baseline was 7.18 cm, and it was 6.47 cm at the last follow-up, indicating a 9.89% reduction in the median SLD during the therapy. The median TV of the target lesions was 124.68 cm^3^ at baseline and decreased to 89.2 cm^3^ at the last follow-up, showing a reduction of 28.46% in the median TV.

### 3.4. Tumor Response Evaluation Based on Administered Treatment

**Figure 8:**
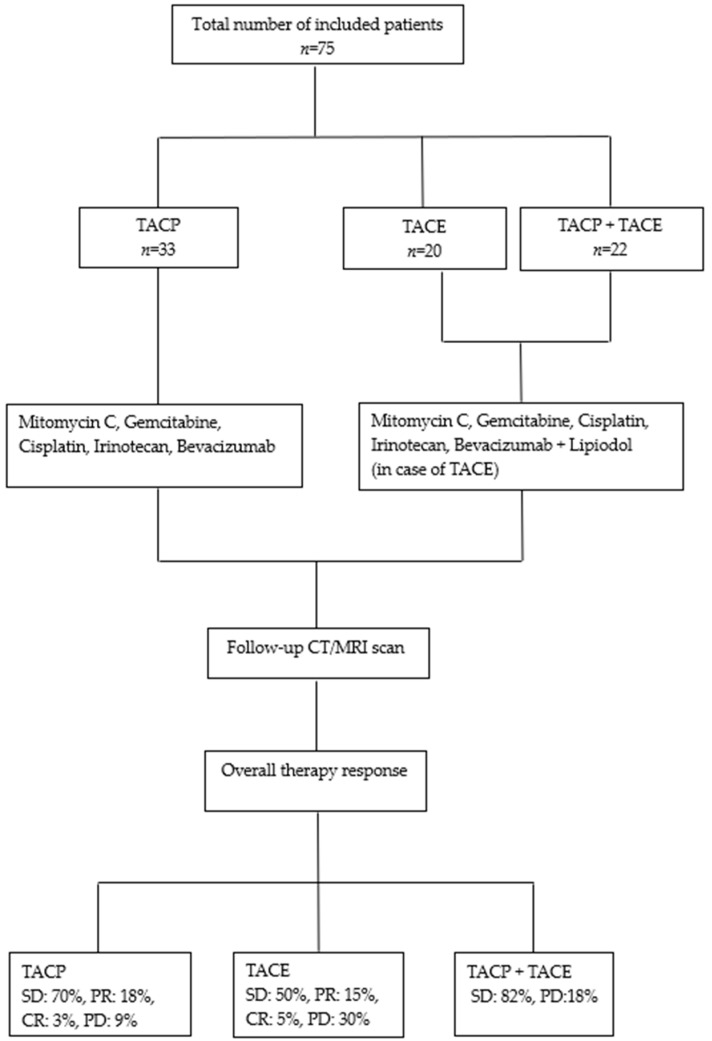
Therapy protocol and tumor response according to RECIST based on the administered treatment.

Upon dividing the patients according to the therapy protocol, we obtained three groups: The first group received only TACP, with an average of 4.7 procedures/patient, and included 33 patients; the second group received only TACE, with an average of 4.9 procedures/patient, and included 20 patients; and the third group received both therapies (TACP + TACE), with an average of 4.7 procedures/patient, and included 22 participants. The results of the tumor response evaluation were as follows (Table 5 and Figure 8): In the TACP group, 23 (70%) of the 33 patients displayed SD, 6 (18%) exhibited PR, 1 (3%) achieved CR, and 3 (9%) experienced PD. A total of 30 patients (91%) exhibited a response to TACP. In the TACE group, 10 (50%) of the 20 participants displayed SD, 3 (15%) exhibited a PR, 1 (5%) had a CR, and 6 (30%) experienced progression. Consequently, 14 (70%) of the patients who underwent TACE responded to the treatment. In the third group, the evaluation showed that 18 (82%) of the 22 participants displayed SD, while 4 (18%) experienced progression. No complete or partial responses were observed in this group.

### 3.5. Survival

**Figure 9:**
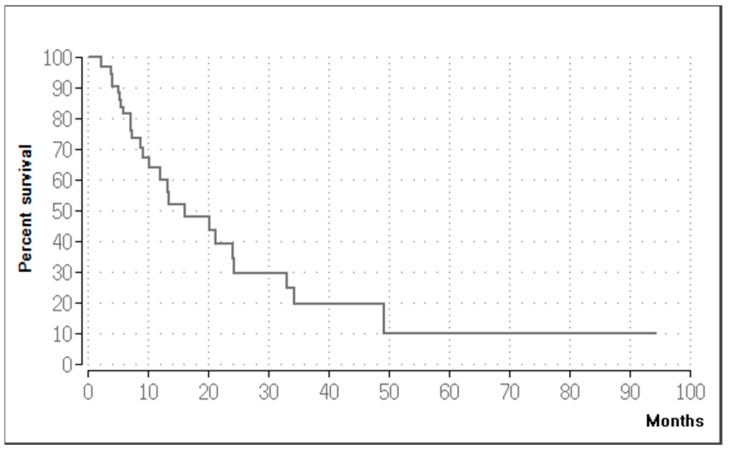
Kaplan–Meier curve of the overall survival (OS) of the entire patient cohort. The median OS was 16.15 months.

**Figure 10:**
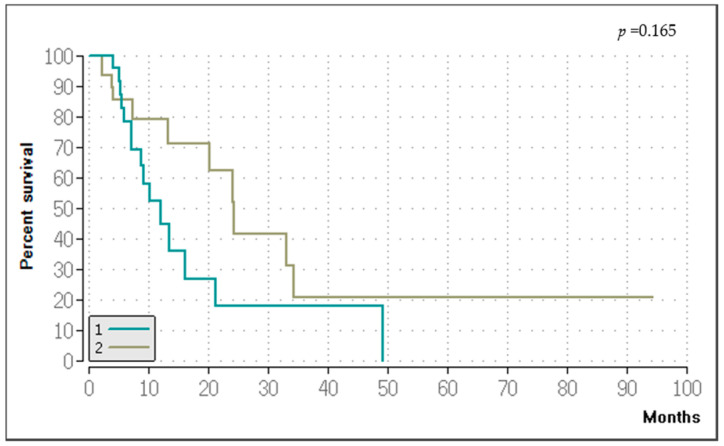
Kaplan–Meier curve of the OS of the patients diagnosed with cervical carcinoma (Group 1, *n* = 36) and of the patients with other gynecologic malignancies (Group 2, *n* = 39). The median OSs were 12 months in the first group and 24.27 months in the second group. The variations in OS between the two groups were not statistically significant (*p* = 0.165).

The Kaplan–Meier method showed that the median OS for the entire patient cohort was 16.15 months, with a 95% confidence interval (CI) of 6–26.3 (Figure 9). The six-month OS rate was 81.5%, the 12-month OS rate was 60.2%, and the 24-month survival rate was 34.5%. One year following the initial procedure, 16 patients were alive. Seven patients were alive after two years, and four patients were alive after three years. The median PFS was 13.19 months (95% CI, 7.8–18.6). When the patients were divided into two groups based on their primary diagnoses, the Kaplan–Meier estimator showed a median OS of 12 months for the cervical carcinoma group (Figure 10) and a median PFS of 8.21 months. The second group had a median OS of 24.27 months and a median PFS of 18.23 months. However, according to the Log-Rank Test, the variations in OS (*p* = 0.165) and PFS (*p* = 0.101) between the two groups were not statistically significant.

## 4. Discussion

The findings of our study suggest that the application of transarterial therapies in the treatment of gynecologic tumors may be an effective means of controlling tumor growth when curative treatments are not available and chemotherapy and radiotherapy are not tolerated or ineffective. Overall, we observed a significant proportion of therapy responders (83%), with the majority (67%) displaying a stable disease. Two patients (3%) exhibited a CR, although this was achieved through the combination of transarterial therapies with thermal ablation. However, further investigation is required to ascertain the curative efficacy of TACE and TACP. Furthermore, a reduction of 6% in the median SLD and a reduction of 8% in the median TV were observed for all 66 patients for whom radiological imaging data were available. Despite the multiple therapeutic interventions that the patients had undergone before participating in our study, we still registered a considerable rate of disease progression (17%). Our findings show that the patients treated with TACP had the highest rate of tumor response (91%) compared to the patients treated with TACE (for whom the response rate was 70%). This difference may be due to the fact that we primarily used TACE for aggressive tumors with a rich arterial supply. Consequently, tumor vascularization may significantly impact the response to transarterial therapies and should be considered an influential factor in treatment outcomes.

Our cohort included a significant proportion of young patients, with 52% of the patients being under the age of 50. The most common malignancy was cervical carcinoma, with 36 cases. Therefore, this group was the focus of our attention. A comparison was made between this group and patients with other gynecologic malignancies, such as ovarian and endometrial carcinoma. The results indicated that transarterial therapies had a greater impact on the heterogeneous group, with a better OS (24.27 months compared to 12 months in the cervical carcinoma group), a better PFS (18.23 months compared to 8.21 months), a reduction in the median SLD and the median TV, and a slightly higher percentage of therapy responders (85% compared to 81%). However, the differences in survival outcomes were not statistically significant. In contrast, other studies have reported higher response rates for cervical carcinoma. For instance, Song et al. [10] evaluated the efficacy and safety of DEB-TACE (drug-eluting bead transarterial chemoembolization) compared with that of CRT for the treatment of advanced cervical carcinoma in 40 patients. The results indicated that there was no significant difference in the treatment response or survival between the two groups. Nevertheless, the tumor response to DEB-TACE was superior to that in our TACE-group: 7 (35%) of the 20 patients had a CR, 9 (45%) had a PR, 3 (15%) had an SD, and only 1 patient (5%) experienced disease progression.

Our results regarding tumor response were superior to those in the study published by Bi et al. [21], in which the safety and efficacy of DEB-TACE in the treatment of advanced or recurrent cervical cancer were evaluated. A reduction in tumor size was observed at three and six months. After 6 months, two of the thirteen evaluated women exhibited an SD (15%), three achieved a CR (23%), two achieved a PR (15%), and six had PD (46%). Therefore, a significant proportion of patients exhibited progression. The study demonstrated a superior OS rate of 19 months in comparison to the OS of 12 months observed in the cervical carcinoma group of our patient cohort. However, our findings demonstrated a superior PFS (8.21 months in our study, and 5.3 months in the other) in a significantly larger patient cohort.

Our results indicate that following transarterial therapy, five patients became eligible for thermal ablation, while one patient became eligible for surgical resection. This suggests that TACP and TACE may be employed as neoadjuvant treatments. However, further investigation is required to assess the efficacy of combining transarterial therapies with thermal ablation in the treatment of gynecologic tumors.

The limitations of this study include its retrospective and single-center design, as well as its varying follow-up intervals and, in many cases, short follow-up period. Furthermore, we had a heterogeneous cohort, encompassing numerous tumor types with varying numbers of patients, posing challenges in presenting the results. Radiological examination was also challenging for the participants who failed to attend their follow-up visits. Additionally, many patients had undergone numerous therapeutic interventions prior to their enrollment in the trial, which may have affected their physical condition and therapeutic response. Furthermore, a heterogeneous treatment strategy was employed, with transarterial therapies for both palliative and neoadjuvant purposes. Consequently, we recommend the implementation of standardized treatment protocols in future trials.

## 5. Conclusions

In conclusion, TACP and TACE are minimally invasive procedures that may be employed as potential treatment options for tumor growth control in patients with unresectable gynecologic tumors. These therapies can be applied in a palliative setting and are particularly beneficial for patients who are intolerant of, exhibit a suboptimal response to, or refuse conventional treatments such as chemotherapy and radiotherapy. Nevertheless, further investigation and adjustment of treatment protocols are recommended to enhance response rates and improve survival outcomes.

## Figures and Tables

**Table 1:** Patients’ characteristics at baseline.

Characteristic	Number
**Age (years)**	
Median	50
Range	24–86
**Primary tumor**	
Cervical carcinoma	36
Ovarian carcinoma	14
Endometrial carcinoma	11
Vaginal carcinoma	6
Vulvar carcinoma	4
Uterine leiomyosarcoma	2
Uterine carcinosarcoma	1
Stromal sarcoma of the ovary	1
**SLD (cm)**	
Median	7.41
Range	1.2–20
**Tumor volume (cm^3^)**	
Median	129.73 cm^3^
Range	4–620 cm^3^

SLD = sum of the longest diameters.

**Table 2:** The median SLD measured at baseline and three-month intervals for each patient group.

Patient Group	Number of Patients	Median SLD at Baseline (cm)	3-Month Follow-Up	6-Month Follow-Up	9-Month Follow-Up
Group 1	46	7.12	6.59	-	-
Group 2	19	6.95	6	5.78	-
Group 3	10	6.99	6.2	6.15	6.42

**Table 3:** The median tumor volume (TV) measured at baseline and three-month intervals for each patient group.

Patient Group	Number of Patients	Median TV at Baseline (cm^3^)	3-Month Follow-Up	6-Month Follow-Up	9-Month Follow-Up
Group 1	46	117	98.48	-	-
Group 2	19	117.27	77.99	68.78	-
Group 3	10	103.4	86	80.87	119.71

**Table 4:** Tumor response evaluation and survival analysis based on the primary diagnosis.

Therapy Response and Survival	Cervical Carcinoma 36 Patients	Other Gynecologic Tumors 39 Patients
Stable disease *n* (%)	26 (72)	25 (64)
Partial response *n* (%)	2 (6)	7 (18)
Complete response *n* (%)	1 (3)	1 (3)
Progressive disease *n* (%)	7 (19)	6 (15)
Therapy responders *n* (%)	29 (81)	33 (85)
Median SLD at baseline	7.58 cm (32 patients)	7.18 cm (34 patients)
Median SLD at the last follow-up	7.6 cm (32 patients)	6.47 cm (34 patients)
Median TV at baseline	135.2 cm^3^ (32 patients)	124.68 cm^3^ (34 patients)
Median TV at the last follow-up	155.7 cm^3^ (32 patients)	89.2 cm^3^ (34 patients)
Median OS	12 months	24.27 months
Median PFS	8.21 months	18.23 months

**Table 5:** Tumor response evaluation based on RECIST for different therapy protocols.

Tumor Response	TACP	TACE	TACP + TACE
Stable disease *n* (%)	23 (70)	10 (50)	18 (82)
Partial response *n* (%)	6 (18)	3 (15)	-
Complete response *n* (%)	1 (3)	1 (5)	-
Progressive disease *n* (%)	3 (9)	6 (30)	4 (18)
Therapy responders *n* (%)	30 (91)	14 (70)	18 (82)
**Total number of patients**	**33**	**20**	**22**

## Data Availability

Due to ethical and privacy reasons, the analyzed data cannot be shared. The publication of this article does not compromise the anonymity of the participants or breach local data protection laws.
